# Measurement of Contractile Stress Generated by Cultured Rat Muscle on Silicon Cantilevers for Toxin Detection and Muscle Performance Enhancement

**DOI:** 10.1371/journal.pone.0011042

**Published:** 2010-06-10

**Authors:** Kerry Wilson, Mainak Das, Kathryn J. Wahl, Richard J. Colton, James Hickman

**Affiliations:** 1 NanoScience Technology Center, University of Central Florida, Orlando, Florida, United States of America; 2 Molecular Interfaces and Tribology Section, U.S. Naval Research Laboratory, Washington D. C., United States of America; 3 Chemistry Division, U.S. Naval Research Laboratory, Washington D. C., United States of America; Sun Yat-Sen University, China

## Abstract

**Background:**

To date, biological components have been incorporated into MEMS devices to create cell-based sensors and assays, motors and actuators, and pumps. Bio-MEMS technologies present a unique opportunity to study fundamental biological processes at a level unrealized with previous methods. The capability to miniaturize analytical systems enables researchers to perform multiple experiments in parallel and with a high degree of control over experimental variables for high-content screening applications.

**Methodology/Principal Findings:**

We have demonstrated a biological microelectromechanical system (BioMEMS) based on silicon cantilevers and an AFM detection system for studying the physiology and kinetics of myotubes derived from embryonic rat skeletal muscle. It was shown that it is possible to interrogate and observe muscle behavior in real time, as well as selectively stimulate the contraction of myotubes with the device. Stress generation of the tissue was estimated using a modification of Stoney's equation. Calculated stress values were in excellent agreement with previously published results for cultured myotubes, but not adult skeletal muscle. Other parameters such as time to peak tension (TPT), the time to half relaxation (½RT) were compared to the literature. It was observed that the myotubes grown on the BioMEMS device, while generating stress magnitudes comparable to those previously published, exhibited slower TPT and ½RT values. However, growth in an enhanced media increased these values. From these data it was concluded that the myotubes cultured on the cantilevers were of an embryonic phenotype. The system was also shown to be responsive to the application of a toxin, veratridine.

**Conclusions/Significance:**

The device demonstrated here will provide a useful foundation for studying various aspects of muscle physiology and behavior in a controlled high-throughput manner as well as be useful for biosensor and drug discovery applications.

## Introduction

Microelectro-mechanical systems (MEMS) have received a great deal of attention in recent years due to their promise for miniaturizing systems for a variety of applications. One particularly alluring facet of MEMS technologies is the possibility of coupling solid state devices with biological components (Bio-MEMS) such as biomolecules, cells, and tissues for creating novel bio-analytical systems. To date, biological components have been incorporated into MEMS devices to create cell-based sensors and assays [1], [2], [3], [4], [5], [6], motors and actuators [7], [8], [9], and pumps [10]. Bio-MEMS technologies present a unique opportunity to study fundamental biological processes at a level unrealized with previous methods. The capability to miniaturize analytical systems enables researchers to perform multiple experiments in parallel and with a high degree of control over experimental variables. This capacity will allow a high throughput approach to studying a wide variety of problems in biology.

One tissue of particular interest with respect to a variety of diseases is skeletal muscle. Diseases affect skeletal muscle in different ways. Some diseases, such as amyotrophic lateral sclerosis (ALS), affect the stimulating inputs from the neuromuscular junction [11]. Other diseases affect the muscle directly such as muscular dystrophy and muscular atrophy [12], which cause deterioration of the muscles' ability to generate force. Thus, it is advantageous to have a system that allows the real-time interrogation of the physiological properties of muscle as well as the controlled addition of exogenous factors for comparative experimentation. However, it is first necessary to be able to apply the measurements to statistical analysis with regard to physiological factors such as peak stress generated, time to peak stress, the time needed for the muscle to relax to half of the peak stress, and the average rate of stress generation [13], [14]. All of these factors give information about the condition of the muscle and can be compared to published values.

The present study outlines a novel method for performing real-time measurements of the physiological properties of cultured skeletal muscle using a Bio-MEMS device. Stresses generated by myotubes were measured using a modified Stoney's equation, which quantifies stresses generated by a thin film on a cantilever with known physical properties [15], [16]. By this method it has been shown that it is possible to quantitatively measure stress on cantilevers that are in agreement with values previously published in the literature for cultured skeletal muscle. This work validates the use of this system as a foundation for a high-throughput Bio-MEMS device.

## Materials and Methods

### Cantilever Fabrication

The layout for the cantilevers was generated using AutoCAD 2004. The patterns were written to chrome coated 5-inch soda-lime glass masks for front and backside photolithography. Cantilevers were fabricated from 6-inch double-sided polished silicon-on-insulator (SOI) wafers with a 5 µm crystalline silicon layer (front side) and a 500 µm silicon dioxide layer (back side). The front side was primed with a 10 nm layer of hexamethyldisilazane (HMDS) to promote resist adhesion. A 5 µm layer of the photoresist AZ 5214 E (Clariant, Muttenz, Switzerland) was spun onto the device layer followed by softbake, alignment, exposure, and development. The device layer was etched using the deep reactive ion etch (DRIE) process at a rate of 2 µm/min. Resist was stripped and a 0.5 µm thick layer of silicon dioxide was deposited via Plasma Enhanced Chemical Vapor Deposition (PECVD) to protect the device layer during subsequent processing. The wafer was then flipped over and was primed with a 10 nm layer of HMDS and spun with 4.15 µm layer of AZ 9245 photoresist (Clariant, Muttenz, Switzerland). Coating was followed by softbake, front-back alignment, development, and DRIE etch at 4 µm/min until the bulk of the back side had been etched through leaving only the buried native oxide layer. The devices were then subjected to a buffered HF dip to remove the buried native oxide layer as well as the silicon dioxide that had been deposited onto the device layer. Individual devices were separated by breaking connecting tabs that were incorporated into the device design. Cantilever dimensions were measured using a JEOL 6400 scanning electron microscope (SEM) at a take-off angle of 50° off normal.

### DETA surface modification

The silicon cantilevers were coated with the amine-terminated alkylsilane, (3-Trimethoxysilyl propyl) diethylenetriamine (DETA) (United Chemical Technologies, Bristol, PA) to promote cell adhesion and differentiation [17]. Cantilevers were cleaned in serial acid baths of concentrated HCl in methanol (1∶1 dilution) for 30 minutes and concentrated H_2_SO_4_ for 1 hr, followed by 30 minutes in boiling de-ionized water. Cleaned cantilevers were dried overnight in an 80°C oven. Surface modification was performed according to a previously published protocol [18]. Briefly, the cantilevers were incubated in 0.1% solution of DETA in toluene for 30 minutes under gentle heating (approximately 70°C), followed by 3X wash in fresh toluene. The coated cantilevers were then heated in fresh toluene for 30 minutes followed by drying overnight in an 80°C oven. Coated samples were stored in a desiccator until use. X-ray photoelectron spectroscopy (XPS) and contact angle measurements were used to characterize the surface coating.

### Cell culture

Skeletal muscle was dissected from the hind limb thighs of a rat fetus at embryonic day 18 (Charles River Laboratories, Wilmington, MA) according to a previously published protocol [18], with some modification. Tissue samples were collected in a sterile 15-ml centrifuge tube containing 1 ml of calcium and magnesium free phosphate buffered saline (PBS). Tissue samples were enzymatically disassociated using 3 ml of 0.05% of trypsin–EDTA (Invitrogen, Carlsbad, CA) solution for 60 min in a 37°C water bath with agitation of 100 rpm. After 60 min, the trypsin solution was removed and 6 ml of L15 media (Invitrogen, Carlsbad, CA) containing 10% fetal bovine serum (FBS) was added to terminate the trypsin action. The tissue was then mechanically triturated using a sterile narrow bore Pasteur pipette, allowed to settle for 3 min, and transferred to a 15-ml centrifuge tube. This was repeated three times. The dissociated tissue was then centrifuged at 300 g for 10 minutes at 4°C on 6 ml of a 4% (wt/vol) cushion of bovine serum albumin (BSA). The pellet was resuspended in 10 ml L15 + 10% FBS and plated in uncoated 100-mm Petri dishes for 20–30 min depending on the amount of tissue, to allow contaminating fibroblasts to settle out. After 20–30 minutes the supernatant was layered on 6 ml of a 4% BSA cushion, and centrifuged at 300 g for 10 min at 4°C. The pellet was resuspended in 1.5 ml of medium.

Purified myocytes were plated at a density of 500–800 cells per square millimeter onto the cantilevers. Myocytes were allowed to attach for 1 hour after which time 3 ml of culture medium (Neurobasal media containing B-27 [Invitrogen, Carlsbad, CA], Glutamax [Invitrogen, Carlsbad, CA], and Pencillin/Streptavidin) was added. Cultures were maintained in a 5% CO_2_ incubator (relative humidity, 85%). Culture medium was exchanged every 4 days. Cantilever/myocyte constructs were allowed to culture for 10–13 days before experiments. During this time myocytes fuse into functional myotubes capable of generating contractile stresses sufficient to deflect the cantilever.

### Detection system setup

**Figure 1:**
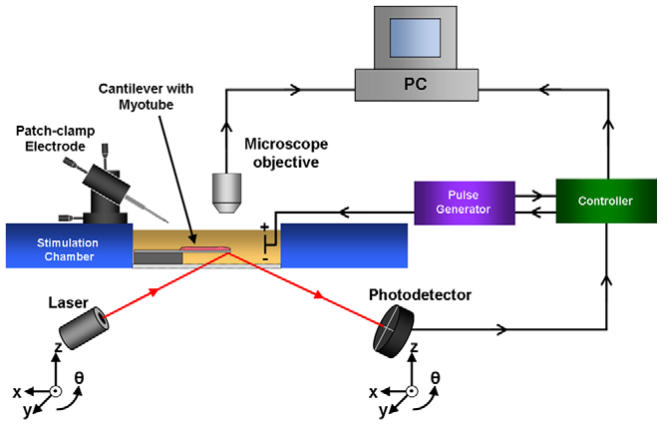
Schematic representation of cantilever deflection system for recording myotube contractions.

A detection system similar to those used in atomic force microscope (AFM) system was designed for measuring deflection of the cantilevers during myotube contraction (Figure 1). The entire system was assembled around an upright Olympus BX51WI electrophysiology microscope (Olympus Inc., Center Valley, PA). The detection system consisted of a class 2 red photodiode laser (Newport, Irvine, CA), a stimulation chamber, a 4-quadrant photodetector (Noah Industries, Melbourne, FL), and a computer with pClamp 10.0 data acquisition software (Molecular Devices, Union City, CA). The laser and photodetector (PD) were mounted on x-y-z-θ translators (Newport, Irvine, CA), which were mounted on the underside of the microscope stage. The stimulation chamber was fabricated from 5 mm thick polycarbonate sheet. An approximately 15 mm×15 mm square chamber was milled out of the sheet and fitted with silver wires (0.015 inch diameter) for field stimulation. The silver wires were mounted parallel to each other with a separation of 15 mm. The bottom of the chamber was sealed using a 22 mm×22 mm glass coverslip. This created a transparent base through which the laser beam could easily pass. The silver wires were connected to an external pulse generator (A–M systems, Sequim, WA) capable of producing field stimulation pulses of varying intensity, frequency, and waveform. Both the pulse generator and PD were connected to an Axon Instruments series 1440 digitizer (Molecular Devices, Union City, CA) which was interfaced with the computer.

### Stress calculation

The stress exerted by a myotube attached along its length to a cantilever can be estimated by considering the system as a cantilever bimorph and using Stoney's equation [15]. Stoney's equation relates the stress in a bimorph system (film on substrate) to curvature of the substrate and the mechanical properties and thicknesses of the substrate and adherent film layer. Stoney's initial assumption of a uniaxial stress in the film has since been updated to describe a biaxial stress to reflect a more accurate representation [16]. Thus we calculate the film stress, σ_film_,using:
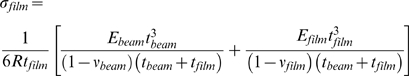
(1)where *E_beam_* and *ν_beam_* are the cantilever material modulus (130 GPa) and Poisson's ratio (0.28), respectively, *t_beam_* is the cantilever thickness, *t_film_* is the myotube thickness, *R* is the effective radius of curvature of the beam caused by the stress in the myotube layer, *σ_film_*.

Many applications of Stoney's formula, most recently for studies of deposited and adsorbed films on thin substrates [19] or cantilevers [16], [20], [21], neglect the second term in the brackets because the films are much thinner than the substrate. In the present Bio-MEMS system, this assumption is not satisfied (*t_film_*∼10 µm compared to the cantilever thickness, *t_beam_*≈5 µm). However, for this system we also neglect this term because the modulus of the myotubes comprising the film on the cantilever are expected to be in the kPa range, at least 6 orders of magnitude lower than the modulus of the beam substrate Si (130 GPa). Thus we write:
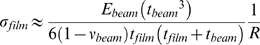
(2)which is known as Atkinson's approximation [22].

When film thicknesses approach or exceed substrate (beam) thicknesses, significant errors can arise when applying Stoney's equation to evaluate film stresses; in particular, the stresses are overestimated when the film is more mechanically compliant than the beam. Klein [23] derived a simple correction factor dependent on modulus ratio, *γ  =  E'_film_/E'_beam_* (where *E'  =  E/(1−ν)*) and thickness ratios *δ =  t_film_/t_beam_* which results in:
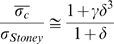
(3)Because in the present experiments *γ*≈0, the correction factor in Eq. (3) can be applied directly to the result from Eq. (2) above, replacing σ_Stoney_ with σ_film_ to obtain *σ*_c_≈σ_film_/(1+*δ*).

The radius of curvature of the cantilever during contraction was calculated using the raw voltage data collected from the PD. This was done taking into account the geometry of the system (path length of the reflected laser, sensitivity of the detector to laser spot position, etc.) [24], [25]. From the raw data the change in angle of the end of the cantilever, θ, was calculated. Using θ it was then possible to calculate the deflection of the free end of the cantilever, *d*, which was then applied to equation 3 to calculate the radius of curvature, R. Experimentally, 1/R is estimated using the measured beam deflection and the geometric approximation:
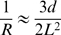
(4)where *L* is the length of the cantilever and *d* is the deflection of the free end of the cantilever [20].

From Figure 1, tensile or compressive stress in the myotube film will result in an upward or downward vertical deflection of the cantilever beam. Measured deflections from the photodiode detector will be reported as positive and negative deflection, *d*, respectively. Since the myotube film is grown on the top face of the cantilever array, deflections due to tensile (positive values) or compressive (negative values) stresses in the film are consistent with standard conventions [26].

### Immunostaining and Confocal Microscopy

After deflection measurements the tissue samples were washed 3X with PBS, then fixed for 15 minutes in 4% (vol/vol) paraformaldehyde at room temperature. Tissues were permeabilized and blocked in a single step using a solution of 0.1% Triton-X100 in PBS, with 10% donkey serum. Blocking and permeabilization was allowed to proceed for 1–2 hours. Afterward, the samples were washed 3X in PBS and incubated with a mouse anti-myosin heavy chain primary antibody (Developmental Studies Hybridoma Bank, Iowa City, IA) overnight at 20°C. Following incubation with the primary antibody, the tissues were washed 3X with PBS and incubated with a donkey anti-mouse secondary conjugated with Alexa Fluor 594 (Invitrogen, Carlsbad, CA) at room temperature for 1–2 hours. The final stained samples were washed again with PBS and imaged under PBS using confocal microscopy.

Myotube thickness was measured by optical sectioning with a Perkin Elmer Ultraview spinning disc confocal microscope (Perkin Elmer, Waltham, MA) under a 40X water immersion lens. The 40X lens was mounted on a piezoelectric z-step motor with a minimum step size of 0.4 µm and a total travel length of 60 µm. Images were collected in 0.5 µm steps from the surface of the cantilever to the top of the tissue. The “z-stack” of images was reconstructed using a 3-D rendering program provided with the microscope. The thickness of the myotube was then measured using the reconstructed image and an internal reference scale.

### Exogenous factors added to muscle culture

In order to demonstrate the usefulness of this device for studying the biology of muscle development and function, experiments were conducted using exogenously applied factors to elicit a measurably different response of the muscle compared to control conditions. The sodium channel agonist veratridine was added to normally cultured myotube on cantilevers and the response was measured with the detection system. After 10 days of culture the myotube/cantilever constructs were placed in the detection system and stimulated with a 1 Hz pulse to elicit synchronous, detectable contractions. Upon confirmation of synchronous contraction, veratridine was added to a final concentration of 5 µM, and the resulting contractile behavior recorded.

Cultures were also performed in order to enhance the contractile capacity of the myotubes. The culture medium NbActiv4 was used *in lieu* of the Neurobasal/B27 formulation used for control cultures. Cantilevers were seeded normally and cultured under conditions identical to those previously stated. After 10–13 days cantilever/myotube constructs were placed in the detection system and stimulated with a 1 Hz pulse train. The calculated values were then compared to previous experiments and published literature.

## Results and Discussion

### Characterization of cantilevers

**Figure 2:**
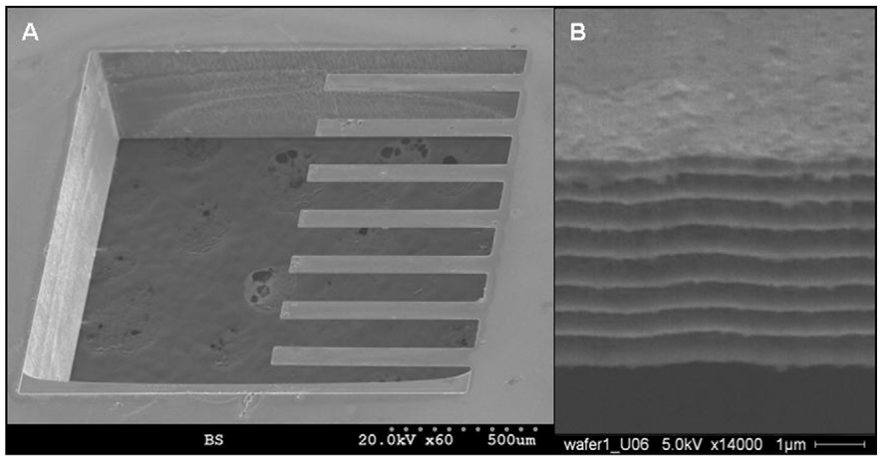
SEM micrographs of silicon cantilevers. A) Low magnification (60x) micrograph of cantilever array. B) High magnification (14000x) image used to measure the thickness of the cantilever. Both images were taken at 50° from normal.

When using Stoney's equation to estimate film stress on cantilevers it is critical to have precise knowledge of the thickness of both the beam and the film. Figure 2 shows representative SEM micrographs of the cantilevers used for the experiments. The cantilevers were measured to have a mean length and width of 755 +/− 3 µm and 109 +/− 1 µm respectively. As shown in Figure 2B the mean thickness of the cantilevers was measured to be 5.27 +/− 0.07 µm. Given these values one can expect a ∼4% error in the stress estimation from experiment to experiment due to variation in beam thickness.

The spring constant of the cantilevers was calculated theoretically and measured experimentally. The calculated spring constant, 1.21 N/m, was determined from the measured dimensions and Young's modulus for crystalline silicon, using the formula for the spring constant of a rectangular cantilever. The spring constant was determined experimentally using the method of Sader et al. [27]. In short the resonant frequency was measured via a ring-down experiment, and the resulting data processed by the spectrum analysis routines in pClamp software. The resonant frequency in air was determined to be 14.3±0.3 kHz. Corrected for damping by air, the resonant frequency of the cantilevers was found to be 14.4±0.3 kHz. This value was then applied to Sader's equation for calculating the cantilever spring constant, which was found to be 1.27±0.06 N/m. Due to the high resonance frequency of the cantilevers, it was expected that the resulting data reflected only the behavior of the myotube contraction as the response time of the cantilevers was on the order of microseconds, whereas the time scale of the muscle contraction was on the order of milliseconds.

### Myotube culture

**Figure 3:**
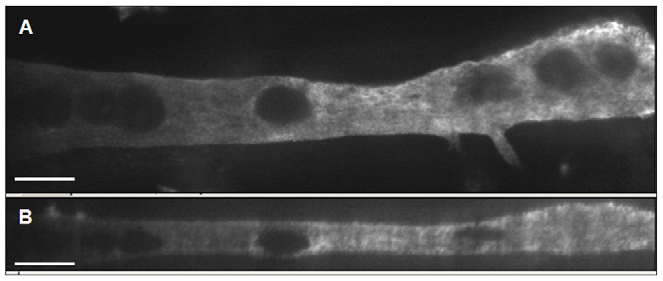
Confocal micrographs of cultured myotubes on cantilevers (cantilever not visible). A) Top down view of a single myotube stained for myosin heavy chain. B) Side view of a three dimensional image reconstructed from a stack of confocal sections. Scale bars are 20 µm.

After plating the dissociated myocytes on the cantilevers, the Bio-MEMS constructs were allowed to culture for 10–13 days during which time the myocytes fused into functional myotubes. Myotube cultures remained viable for as long as 4 weeks, however, experiments were performed at 10–13 days due to some variability associated with maintaining long-term cultures. However, the culture system has now been shown to be viable for a long as 90 days in culture, so longer term experiments are possible [28]. During the fusion and differentiation process the myotubes spontaneously oriented along the long axis of the cantilever, facilitating bending of the cantilever. It should be noted, however, that the orientation of the myotubes was not always directly parallel to the long axis of the cantilever. This configuration resulted in some torsional bending, and hence a possible underestimation of the total contractile stress. Typically the coverage of myotubes on cantilevers was greater than 95%. Occasionally, tissue coverage was less due to tissue processing, suboptimal surface modification, or other systematic errors. Only robust cultures with morphologically normal looking myotubes were used for deflection experiments. Figure 3 shows a confocal microscope image of a section of a representative myotube cultured for 13 days on a DETA modified cantilever (not visible). Figure 3A shows the top down projection of the z-stack in the plane of the cantilever. The data from the z-stack of images were reconstructed into a 3-dimensional representation of the myotube geometry. Figure 3B is a side view showing the thickness of the myotube along a section of the cantilever. The mean thickness of the myotube was ∼10 µm. Due to the morphology of the myotube, however, the thickness was not necessarily uniform throughout the length of the cantilever. The thickness has been measured to range between 5 µm to 15 µm on individual cantilevers. This variation in film thickness throughout the tissue can potentially lead to discrepancies between true and calculated stress values. In this study we used the average value of 10 µm for calculations. The effect of the thickness variation on the calculated stress will be considered in a later section.

### Stress Calculation

**Figure 4:**
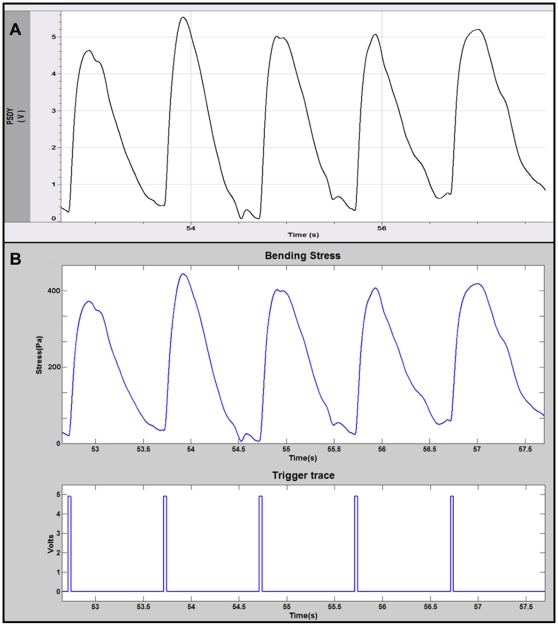
Stress calculation using the modified Stoney's equation. A) Raw data measured from the cantilever deflection system. B) Estimated stress calculated using the modified Stoney's equation.

Figure 4 shows both the raw voltage data from the PD and the resulting stress calculated using the modified Stoney's equation. Figure 4A shows the raw data collected in free-run mode from myotubes cultured for 13 days and stimulated with a 5 volt DC pulse at a frequency of 1 Hz. As shown previously [29] this allowed selective stimulation of the myotubes to actuate the cantilevers. Each trigger pulse, Figure 4B, corresponds precisely with the onset of a myotube contraction. The myotubes responded to the stimulation in a frequency dependent manner; increasing or decreasing the stimulation frequency resulted in a corresponding change in the frequency of myotube contraction (data not shown). As with previously published results, stimulation at or above a frequency of 10 Hz induced a state of fused tetanus [29]. Figure 4B indicates the results from Stoney's calculation using the raw data. The stresses calculated from this data set ranged between 0.4 kPa and 0.5 kPa. These values are in excellent agreement with previously published literature for cultured skeletal muscle [30], which reported average peak twitch stress values of 2.9 kPa (reported as specific peak twitch force in units of kN/m^2^), but less than 1% of those expected for adult muscle, ∼300 kPA [14], [30]. This is not surprising due to the fact that the tissue used in this study was collected from embryonic stage rat pups and cultured *in vitro* for only 13 days after dissection. It is possible that the culture conditions, as published here, were not sufficient for the development of myotubes with adult phenotype. Similar observations were made by Dennis and coworkers [30] for cultured adult rat myoids, noting the possibility of developmental arrest in culture that prevented the development of adult isoforms of myosin.

**Figure 5:**
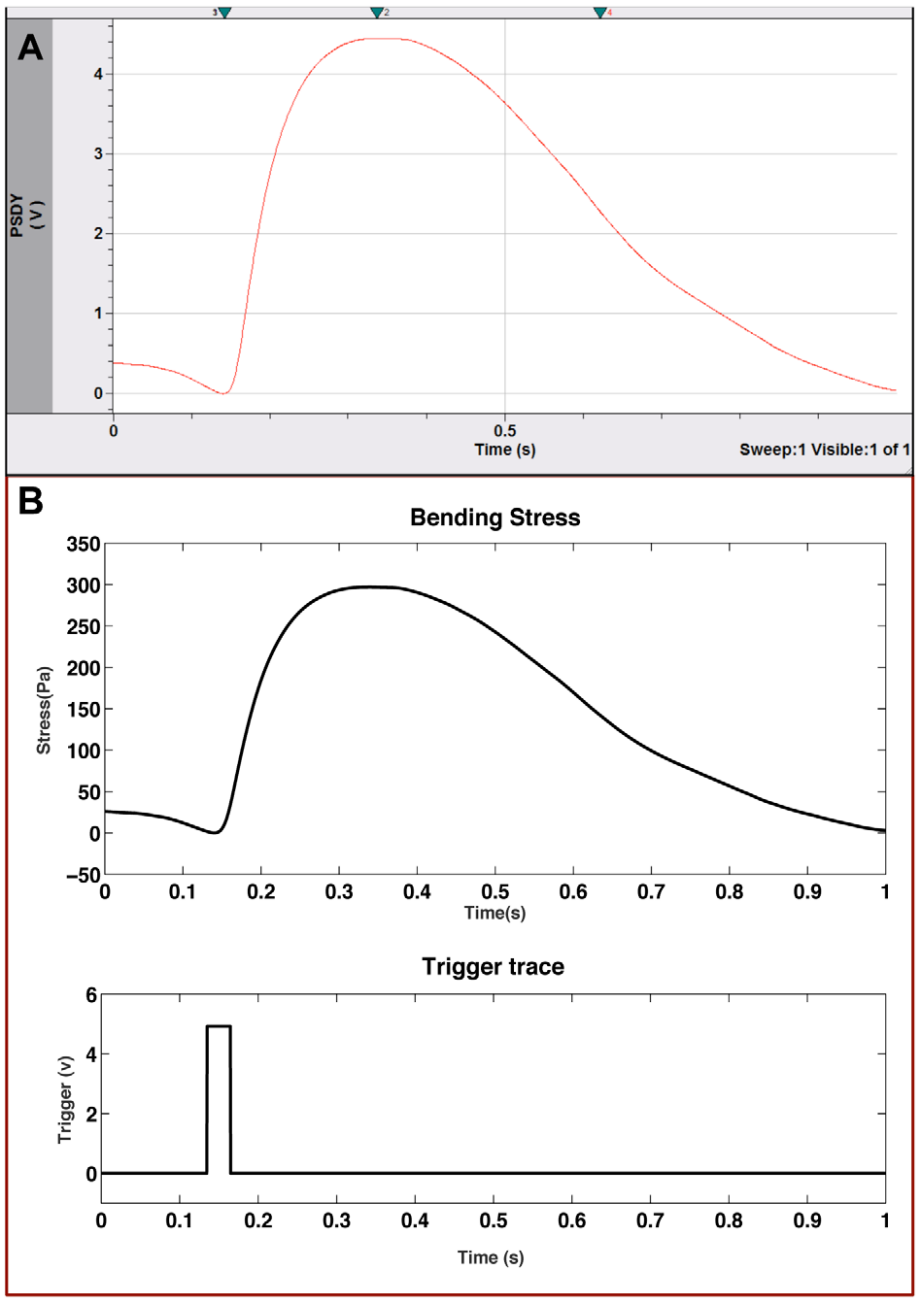
Averaged data from 11 myotube contractions. A) Raw data, B) Estimated stress calculated using the modified Stoney's equation.

To further characterize the myotubes, two other parameters were analyzed: the time to peak twitch stress (TPT), which is the time required to reach stress from the onset of contraction, and time to half relaxation (½RT), which is the time required to relax to 50% of peak tension. Figure 5 shows both the raw data (Figure 5A) and calculated stress averaged from 11 myotube contractions (Figure 5B). The resulting average peak contractile stress for these data is ∼1.1 kPa, which is in agreement with previously stated results. The calculated values for TPT, and ½RT (Table 1) were significantly longer than those published for cultured muscle by Dennis and coworkers [30] and for adult rat muscle as published by Close [13]. The average TPT for the cultured myotubes was 236.8±26.1 ms. This value is considerably slower than that of 69.3±9.4 ms published by Dennis for cultured rat myoids as well as values of 65.0±3.8 ms and 36.0±2.3 ms, for neonatal and adult rat respectively, published by Close. The ½RT values for cultured myotubes were also prolonged compared to those reported by Dennis et al. and Close. The average ½RT for the data presented in Figure 5 was measured to be 233.6±23.8 ms. Dennis reported ½RT values of 116.4±19.4 ms for myoids, while Close reported values of 70.0±4.9 ms for neonatal muscle and 48.0±3.4 ms for adult muscle. It is interesting to note, however, that the TPT:½RT (∼1∶1) ratio for the cultured myotubes was closer to that of the neonatal and adult rat muscle than that of the cultured myoids (∼1∶1.7). It can be concluded from these data that the myotubes cultured in the Bio-MEMS system, while exhibiting contractile stress magnitudes comparable with those previously published for cultured rat muscle, show evidence of a more embryonic phenotype with regard to other important physiological parameters. This is further reinforced by previously published results that showed staining of similarly cultured myotubes for embryonic myosin heavy chain, but not the adult or fetal isoforms [31].

**Table 1:** Average values for stress, TPT, and ½ R_t_ from embryonic skeletal muscle (ESM), and previously published work from Dennis and Close.

	σF (kPa)	TPT (ms)	½ RT (ms)	dσ/dt (Pa/ms)
ESM	∼0.3	236.8±26.1	233.6±23.8	2.4
Dennis and Kosnik (2000)	∼1.0	69.3±9.4	116.4±19.4	75.3
Close (1964)	>300	36.0±2.3	48.0±3.4	N/A

### Variation in Stress Calculation due to film thickness

**Figure 6:**
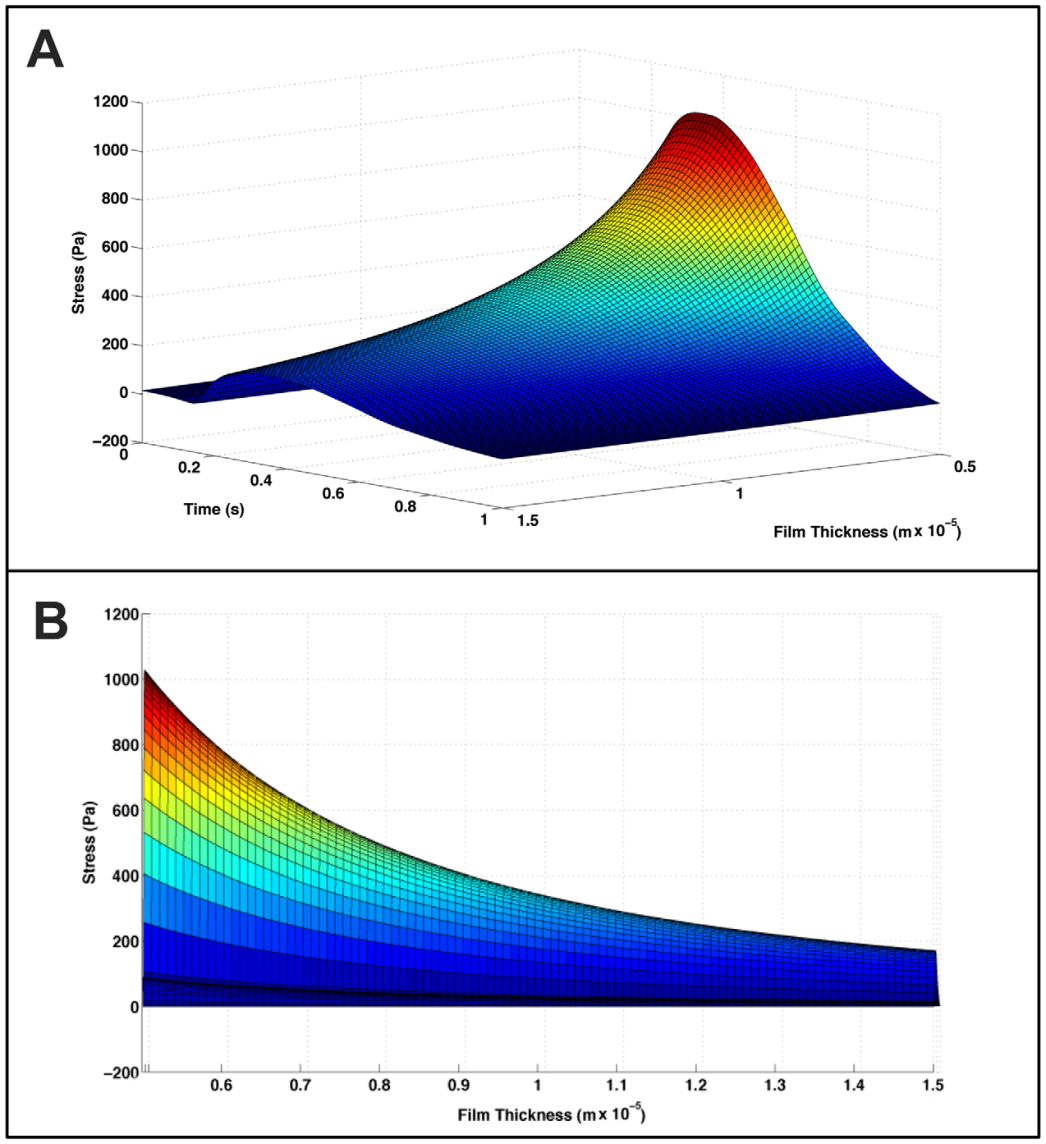
Variation in stress vs. film thickness. A) Data from Figure 5 plotted vs. film thickness. B) Peak contractile stress plotted vs. film thickness.

As previously stated, the thickness of the myotube film on the cantilever has been measured to vary between 5 to 15 µm (Figure 3). Figure 6 shows the variation in calculated stress due to film thickness. Figure 6A is a plot of the variation in calculated stress, using the same data shown in Figure 5A, as a function of film thicknesses ranging from 5 to 15 µm. It is clear from this graph that there is a significant variation in the calculated stress due to the measured film thickness. Figure 6B indicates a plot of the calculated peak contractile stress vs. film thickness. In this plot it can be seen that the stress values range from ∼0.2 kPa to ∼1.0 kPa over the selected film thickness values. It is interesting to note that the Stoney's calculation is particularly sensitive to variations in the film thickness in the range encountered here. Below 5 µm the stress values increased exponentially. Above 15 µm the change in stress due to film thickness slowed considerably. This reinforces the need for accurate measurements of the myotube thickness and standardization of the culture methods to minimize variations of the same. It should be noted, however, that even though there is obviously significant variation in calculated stress these values are still within the range of those published by Dennis et al. (0.9 kN/m^2^ to 5.0 kN/m^2^). These results validate this approach as a method for measuring contractile stress generated by cultured skeletal muscle in a Bio-MEMS device.

### Addition of exogenous factors modulates function of cultured myotubes

#### Addition of the sodium channel agonist veratridine

**Figure 7:**
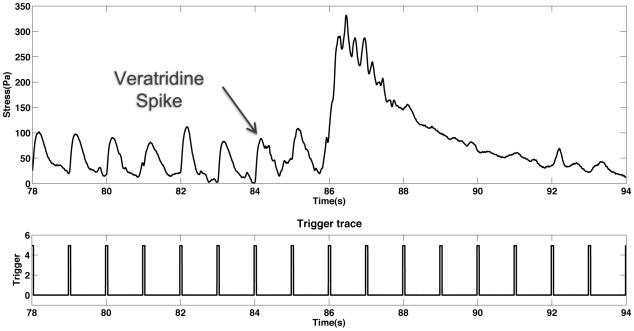
Contractile myotubes were exposed to the sodium channel agonist veratridine. Myotubes were contracting synchronously with the 1 Hz stimulus when, at 84 seconds into the recording, veratridine was injected. After injection of the toxin the muscle tissue contracted in a tetanic manner, and lost the ability to contract further.

Given the ability of this method for quantifying muscle contraction stress and dynamics in real-time, it is ideal for studying the effect of exogenously applied factors on muscle physiology. One such example was the addition of the toxin veratridine to the stimulation chamber during electrical stimulation. Veratridine is an agonist that causes the persistent opening of voltage-gated sodium channels. Normally, upon depolarization of the cell membrane from either field stimulation or neurotransmitter release from neural inputs, voltage-gated sodium channels open allowing an influx of sodium ions into the cytoplasm which further depolarizes the sarcoplasmic reticulum causing calcium release and contraction. After a certain refractory period the voltage-gated sodium channels close and the resting membrane potential is restored. Veratridine binds to the voltage-gated sodium channels causing an abnormally high release of sodium into the cytoplasm, tetanic contraction, and if it is not removed, cell death. Figure 7 shows a recording from contracting skeletal muscle before and after the addition of veratridine. Before addition the muscle was contracting normally in synchronization with the one Hz stimulus. At 84 seconds veratridine was injected into the stimulation chamber and allowed to diffuse to the tissue. As seen in Figure 7 upon injection of the veratridine the muscle began to contract in an asynchronous, tetanic manner with a peak stress far beyond those of the synchronized contractions. After the initial tetanic contraction, the muscle then lost the ability to further contract and the stress exerted on the cantilever returned to baseline. This is the reaction that is expected upon exposure to this toxin. It should be noted that to fully evaluate the usefulness of this system for toxicology studies, dose-dependent experiments should be performed to determine the response of the system over a range of toxin concentrations. Future work will expand on this proof-of-principle experiment using multiple factors over a range of concentrations.

#### Growth of myotubes in NbActiv4 to enhance muscle contractility

**Figure 8:**
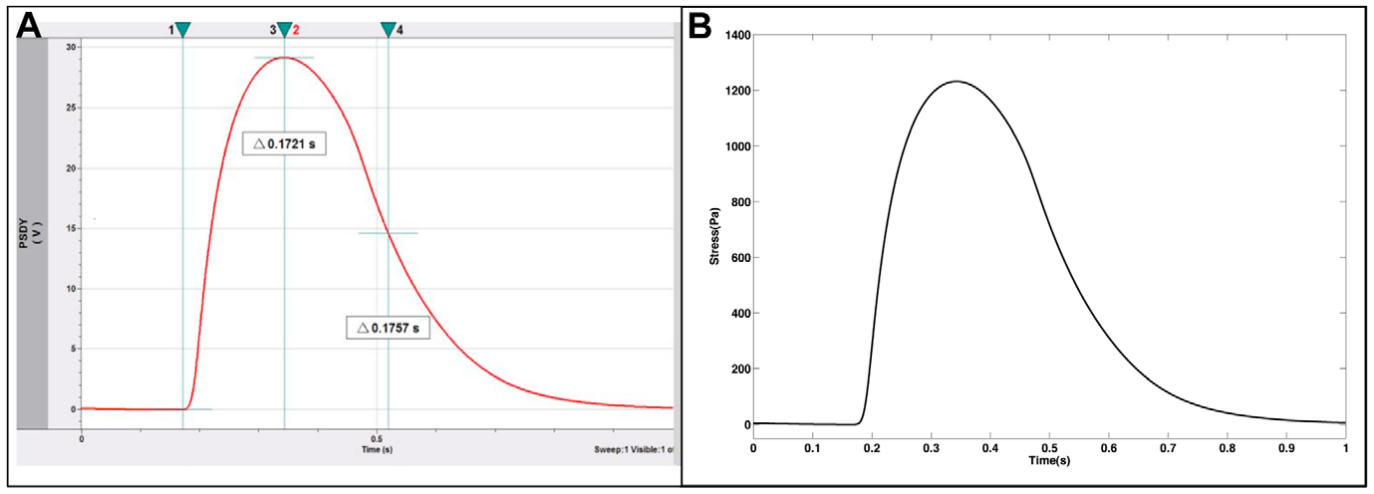
Contraction kinetics from muscle tissue cultured in NbActiv4 media. A) raw data recorded from Bio-MEMS device showing TPT and ½ RT, B) Stress values calculated using the modified Stoney's equation.

As stated in previous sections, the contractile phenotype of the muscle cultured in this system was of an embryonic nature. For this device to serve as a model system for the study of normal muscle it is necessary to be able to culture muscle of a more adult phenotype. To accomplish this it was necessary to supply additional factors that promote the development of more mature contractile properties in the myotubes. The culture medium NbActiv4 is a proprietary formulation based on Neurobasal medium and the growth factor cocktail B27 [32]. NbActiv4 contains three additional growth factors (creatine, cholesterol, and estrogen) that have been shown to produce an eight-fold increase in spike activity in cultured neurons. However, these extra growth factors are also critical for the development of the contractile mechanism of skeletal muscle. For this reason cultured embryonic skeletal myotubes were grown on silicon cantilevers in NbActiv4 to quantify the changes in myotube development due to the added growth factors. Figure 8 shows representative contraction data for a myotube/cantilever system. Figure 8A shows the raw data recorded by the photodiode for NbActiv4 cultured muscle as well as the calculated stress values in Figure 8B. Here it can be seen that the TPT measured was 172.1 ms and the ½ RT 175.7 ms.

Table 2 indicates the comparison of the NbActiv4 cultured muscle with previously published results as well as myotubes cultured in Neurobasal/B27. It can be seen that the addition of NbActiv4 enhances the contractile properties of the myotubes significantly. Most notably the contractile stress generated by NbActiv4 myotubes, 1.2 kPa, is approximately 4 fold higher than those cultured in Neurobasal/B27, which was 0.3 kPa. Although this value is still much less than the stress generated by adult muscle, it is comparable to that published by Dennis et al [30]. It should be noted that while increased contractile stresses were measured, the average thickness of the myotube layer in the NbActiv4 media was not found to vary significantly from those of the control experiments. Also, TPT and ½ RT values for NbActiv4 myotubes decreased significantly compared to muscle cultured in Neurobasal/B27. This decrease in contraction time demonstrates that the myotubes were driven down a path towards a more mature phenotype, in the process developing fast-twitch isoforms of myosin, while increasing the speed of contraction. A detailed immunocytochemical and morphological characterization of the effect of NbActiv4 on developing muscle was performed by Das and coworkers [28] and supports this observation. Furthermore, the increase in average stress generation (dσ/dt) by five fold reinforces the argument that the contractile apparatus of myotubes grown in NbActiv4 is more mature and capable of greater stress generation.

**Table 2:** Contractile properties of NbActiv4 cultured muscle versus previous results and published literature.

	σF (kPa)	TPT (ms)	½ RT (ms)	dσ/dt (Pa/ms)
ESM	∼0.4	236.8±26.1	233.6±23.8	2.4
NbActiv4	∼1.3	172.1±4.7	175.6±3.6	11.8
Dennis and Kosnik (2000)	∼2.9	69.3±9.4	116.4±19.4	75.3
Close (1964)	>300	36.0±2.3	48.0±3.4	N/A

Values for dσ/dt were not available in Close, but average force generation has been reported to be more than 1000 fold higher than that measured in Dennis and Kosnik.

The work presented in this manuscript demonstrates the development of a novel Bio-MEMS device for studying skeletal muscle and its development using microfabricated silicon cantilevers and alkylsilane surface chemistry. The usefulness of this device has been demonstrated for real-time interrogation of cultured skeletal muscle and the quantification contractile stress and kinetics. It has also been shown that physiological phenomena such as tetanus, and response to exogenously applied factors can be monitored and quantified.

Cultured myocytes spontaneously differentiate into functional myotubes on silicon cantilevers coated with DETA that produce contractile stress sufficient to deflect the cantilevers. By applying electrical field stimulation, it was possible to selectively actuate the myotubes on cantilevers in a frequency and intensity dependent manner. This ability to selectively actuate a cantilever is advantageous as it allows a high degree of control over the timing and nature of contraction. This method could also be applied to create bio-robotic devices using skeletal muscle as on actuator on a microfabricated device. Previous studies have utilized cardiomyocytes to provide mechanical force. However, cardiac tissue contracts in a primarily spontaneously manner unlike skeletal muscle which will remain inactive in the absence of stimulating inputs. Also, skeletal muscle is preferable over cardiac muscle due to its rate-response characteristics. As stimulation frequencies increase, contraction frequency and force generation of skeletal muscle will also increase until tetanus is induced, resulting in tonic contraction. Cardiac muscle, on the other hand, will cease to contract under high frequency stimulation, a situation similar to that of cardiac infarction.

Furthermore, it has been shown that neuronal cell types can be patterned and cocultured with skeletal muscle using surface chemistry and microfluidics and that neuromuscular junctions can be found in the system [33], [34]. The ability to create organized neural/muscle cocultures will enable the creation of *in vitro* biological circuits that can be used for a variety of applications (pharmacology, basic science, biorobotics/bioprosthetics). Still it must be understood that many technological hurdles remain to be overcome to realize the full potential of this technology.

This technique holds particular promise for applications in drug discovery and as a model for various diseases involving skeletal muscle, especially since more developmentally mature forms of the myotube culture can now be maintained for up to 90 days in this system [18]. The development of an *in vitro* model for functional biological circuits would greatly benefit the broader scientific community and society in general. By creating lab-on-chip systems that allow high-throughput, real-time experimentation, research costs would be reduced, data collection and analysis would be simplified, and the need for costly and ethically questionable animal studies would be reduced.

### Conclusion

The present work demonstrates a novel method for quantitatively measuring the contractile stress generated by functional cultured skeletal muscle using a Bio-MEMS device, which can selectively stimulate the contraction of a myotube and simultaneously record the physiological behavior in real time. It has been shown that it is possible to culture functional embryonic skeletal myotubes on silicon cantilevers that possess contractile strengths comparable to those previously published for cultured skeletal muscle, although they differ in other important physiological parameters. From these data it is possible to draw conclusions about the developmental state of the tissue when compared with previously published data.

While using the modified Stoney's equation has allowed the calculation of contractile stresses in this system, it is based on an assumption of an average thickness in an inhomogeneous film. Stress values calculated using t_film_ values over the range of those measured still yield numbers that fall within the range of previously published results. Calculation of the more complex problem of stress of cells with variable thickness on cantilevers will likely require numerical methods such as finite element analysis. The validation of this approach will ultimately allow the realization of a high-throughput Bio-MEMS device for studying the basic biology of skeletal muscle and its related pathologies.
